# The impact of peripheral neuropathy symptoms, self-care ability, and disturbances to daily life on quality of life among gynecological cancer patients undergoing chemotherapy: a cross-sectional survey

**DOI:** 10.4069/kjwhn.2022.11.21

**Published:** 2022-12-29

**Authors:** Sohee Mun, Hyojung Park

**Affiliations:** 1 Department of Nursing, Asan Medical Center, Seoul, Korea; 2 College of Nursing, Ewha Womans University, Seoul, Korea

**Keywords:** Activities of daily living, Genital neoplasms, female, Peripheral nervous system diseases, Quality of life, Self care

## Abstract

**Purpose:**

This study investigated the effects of peripheral neuropathy symptoms, self-care ability, and disturbances to daily life on quality of life (QoL) among gynecological cancer patients undergoing chemotherapy.

**Methods:**

The participants included 144 patients with gynecological cancer undergoing anticancer chemotherapy at a tertiary hospital in Seoul, South Korea, from December 1, 2021 to January 28, 2022. Convenience sampling was used to recruit patients who had received 4 or more cycles of chemotherapy using a paclitaxel-platinum regimen, and a self-reported questionnaire was used to collect data. Descriptive statistics, the t-test, analysis of variance, Scheffé test, Pearson correlation coefficients, and multiple regression analysis were performed.

**Results:**

Most of the participants had ovarian cancer (70.1%) or endometrial cancer (14.6%), and the most common number of treatment cycles was 6 to 10 (29.2%). The mean QoL (60.83±19.89) was greater than the midpoint. The regression model analyzing the patients’ QoL was statistically significant (F=15.38, *p*<.001) with an explanatory power of 56.7%. Self-care ability (β=.39, *p*<.001), disturbances to daily life (β=–.38, *p*<.001), the duration of peripheral neuropathy symptoms (β=2.14, *p*=.036), and regular exercise (β=–2.12, *p*=.04) were found to significantly affect QoL.

**Conclusion:**

Efforts to improve the self-care ability of gynecological cancer patients who have experienced peripheral neuropathy after receiving chemotherapy and mitigate disturbances to their daily life can improve their QoL. Healthcare professionals should identify peripheral neuropathy symptoms and examine the effects of the symptoms on patients’ daily lives. Improving the self-care ability of patients and alleviating their limitations in daily life may improve QoL.

## Introduction

Due to advances in medical technology, physicians are increasingly able to diagnose and treat cancer early and properly, and the survival rate of cancer patients is subsequently increasing, with an estimated global number of cancer survivors of 19.3 million people as of 2020 [1]. If South Korean citizens survive until their life expectancy, the probability of developing cancer is 37.4%, and an estimated 2 million cancer patients in Korea have at least one of 24 cancer types as of 2018. Between 2014 and 2018, the 5-year survival rate of gynecological cancer was 65.2% for ovarian cancer, 88.6% for endometrial cancer, and 80.5% for cervical cancer, which indicates that a substantial number of patients survive cancer [2]. Cancer survivors experience problems related to complications. In particular, many patients who undergo chemotherapy suffer from side effects such as nausea, vomiting, alopecia, peripheral neuropathy, and infection [3] and experience chronic side effects, fatigue, and deteriorated quality of life (QoL) after treatment [4]. In addition to the physical side effects caused by the treatment process, patients experience changes in their roles in their families, psychological problems such as anxiety and depression about cancer recurrence, and limitations to social activities, which have negative impacts on QoL [5]. Moreover, general characteristics including age, income quartile, and subjective health status and disease-related characteristics such as limitations to activities, depression, fatigue, pain, anxiety, resilience, and support were also found to affect the QoL of cancer patients [6-9].

In particular, the factors that affect the QoL of gynecological cancer patients include lower limb lymphedema, depression, sleep disorders, recurrence, peripheral neuropathy, distress, changes in subjective health status, and physical changes [9-12]. The use of neurotoxic anticancer drugs to treat gynecological cancer patients is a major factor that causes peripheral neuropathy [13], the symptoms of which can last a long time even after chemotherapy ends, leading to disturbances to patients’ activities of daily living [14]. Since the recurrence rate of ovarian cancer is 70%, most patients with the disease undergo chemotherapy multiple times, which worsens peripheral neuropathy symptoms [15]. Repeated chemotherapy negatively affects the physical and social functions of gynecological cancer patients [16], and psychological problems such as anxiety and depression caused by physical changes including edema and alopecia negatively affect their QoL [17].

Although lymphedema experienced by gynecological cancer patients also causes difficulties related to activities of daily living [9], persistent peripheral neuropathy symptoms lead to both functional and sensory impairment, which further limits patients’ activities of daily living [14]. Simple activities such as walking and dressing may be restricted due to decreased muscle strength and loss of balance [18], and as the symptoms of neuropathy worsen, QoL and functional independence are also impaired. As gynecological cancer patients continue to undergo chemotherapy, their symptoms of peripheral neuropathy worsen and various physical functions become impaired, affecting QoL due to disturbances to activities of daily living. However, a high self-care ability correlates to the proper management of various symptoms experienced by patients in the treatment process, leading to improved QoL [19]. In a study of female cancer patients, a higher self-care ability also correlated to a higher QoL [20], and in an international study, a higher self-care ability was found to lead to proper symptom management and improved QoL [21]. Self-care ability is a major factor that can help cancer patients return to normal life after receiving treatment, raise their functional independence, and improve their QoL.

Few studies, however, have analyzed the effect of self-care ability on QoL among gynecological cancer patients. While previous studies analyzing the QoL of gynecological patients have mainly investigated psychological factors [10-12], further efforts should be undertaken to analyze and understand the effect of physical restrictions on QoL so that patients can better return to normal life after treatment and improve their QoL.

Prior studies showing that the disease-related characteristics and restricted activities of gynecological patients are factors that affect QoL and the theoretical basis that improved self-care ability can raise QoL were considered in this study. Therefore, we attempted to identify the relationship between peripheral neuropathy symptoms, disturbances to daily life, self-care ability, and QoL among gynecological cancer patients and provide basic data for the development of intervention programs to improve the QoL of gynecological cancer patients.

The purpose of this study was to identify the impact of peripheral neuropathy symptoms, self-care ability, and disturbances to daily life on the QoL of gynecological cancer patients undergoing chemotherapy. The specific purposes were as follows:

(1) Analyze the peripheral neuropathy symptoms, self-care ability, disturbances to daily life, and QoL of gynecological cancer patients

(2) Identify differences in the QoL of gynecological cancer patients according to their general characteristics and disease-related characteristics

(3) Identify the relationships between peripheral neuropathy symptoms, self-care ability, disturbances to daily life, and QoL in gynecological cancer patients

(4) Identify the factors that influence the QoL of gynecological cancer patients

## Methods

Ethics statement: This study was approved by the Institutional Review Board of Asan Medical Center (2021-1563). Informed consent was obtained from the participants.

### Study design

This is a correlational study investigating the relationship between peripheral neuropathy symptoms, self-care ability, disturbances to daily life, and QoL of gynecological cancer patients undergoing chemotherapy. This study was written according to the STROBE reporting guidelines (https://www.strobe-statement.org).

### Participants

This study analyzed data on gynecological cancer patients undergoing chemotherapy at Asan Medical Center in Seoul, South Korea. The inclusion criteria were (1) adults aged 19 years or above, (2) diagnosed with gynecological cancer (ovarian, endometrial, cervical, vaginal, or vulvar cancer), and (3) participants who received four or more concurrent cycles of paclitaxel and platinum-based drugs, basd on a study that showed peripheral neuropathy frequently occurs after four or more doses of paclitaxel and platinum-based drugs. The exclusion criteria were (1) participants who experienced peripheral neuropathy before undergoing chemotherapy and (2) participants for whom the exact reasons for peripheral neuropathy were unknown due to spinal and brain metastases.

The sample size for this study was calculated using G*Power 3.1.9. Based on a prior study [18], the minimum sample size needed for multiple regression analysis with a significance level of .05, power of .95, median effect size of .15, and four independent variables (peripheral neuropathy symptoms, the duration of peripheral neuropathy symptoms, self-care ability, and disturbances to daily life) was 129 participants. Questionnaires were distributed to 144 participants, considering a possible 10% dropout rate. Questionnaires were collected immediately after distribution and the collection rate was 100%, and 144 questionnaires were used for the analysis.

### Instruments

Permission was obtained from the developer and adapter before using any of the instruments included in this study.

#### Quality of life

QoL was evaluated using the Korean version of the Functional Assessment Cancer Therapy-General developed by Cella et al. [22] for measuring the QoL of cancer patients. The tool evaluates a total of 27 items, including seven items on physical state, seven items on social/family status, six items on emotional state, and seven items on functional state, and all regular items are scored on a scale ranging from 0 (strongly disagree) to 4 (strongly agree), while reverse items are scored on a scale ranging from 0 (strongly agree) to 4 points (strongly disagree). A higher score corresponds to a higher QoL, with possible scores ranging from 0 to 108. The reliability of the instrument as indicated by Cronbach’s α was .89 in the original study [22], .86 in a developmental study using the Korean version [23], and .84 in this study.

#### Peripheral neuropathy symptoms

Peripheral neuropathy symptoms were evaluated using an instrument developed by Tofthagen [24] and translated and verified by Hwang and Park [25]. Nine items on the occurrence of peripheral neuropathy symptoms, six items on the scope of occurrence, and nine items on the intensity of the symptoms were used in this study. For the intensity of the symptoms, nine items are evaluated on a 10-point scale (0 points, no symptoms; 10 points, very severe). A higher score corresponds to more severe symptoms, and possible scores range from 0 to 90 points. The reliability of the instrument as indicated by Cronbach’s α was .94 in the original study [24], .92 in a translated study [25], and .91 in this study.

#### Self-care ability

Self-care ability was evaluated using an instrument developed by Geden and Taylor [26] and revised and translated into Korean by Jung [27]. It contains 32 items and uses a 6-point Likert scale (1 point, strongly disagree; 6 points, strongly agree). A higher score corresponds to a higher self-care ability, and possible scores range from 32 to 192 points. The reliability of the instrument as indicated by Cronbach’s α was .96 in the original study [26], .92 in a translated study [27], and .98 in this study.

#### Disturbances to daily life

Disturbances to daily life were evaluated using an instrument developed by Tofthagen et al. [24] and translated into Korean and verified by Hwang and Park [25]. Fourteen items that measured disturbances to daily life were used in this study and scored based on a scale ranging from 0 points (no effect at all) to 10 points (strong effect). A higher score corresponded to a higher degree of disturbances to daily life, and possible scores ranged from 0 to 140 points. The reliability of the instrument as indicated by Cronbach’s α was .94 in the original study [24], .92 in a translated study [25], and .94 in this study.

#### General characteristics and disease-related characteristics

General characteristics included six items on age, marital status, education level, economic status, living with family, and regular exercise, and disease-related characteristics, which included type of cancer, the first instance of chemotherapy-induced peripheral neuropathy (CIPN), and the duration of CIPN (3 items), were investigated using a structured questionnaire. In addition, three further items were investigated using electronic medical records and included the number of types of chemotherapy the patient had undergone, the number of chemotherapy treatments received, and the cumulative amount of anticancer agents administered.

### Data collection

Data were collected using the convenience sampling method to identify patients undergoing chemotherapy in the gynecological ward and outpatient injection room of the hospital from December 1, 2021 to January 28, 2022. The researchers explained the purpose and procedures of the study and ensured the confidentiality of the data to the participants in person, after which their consent to participate in the study was obtained. Data were collected using questionnaires that were completed by the participants themselves. If it was difficult for patients to complete the questionnaire, the researchers read the questions aloud to the participants and recorded their answers. The questionnaire took approximately 20 minutes to complete, and the participants were provided with a small gift (KN95 masks) upon completion. After the questionnaires were completed, disease-related items were collected from electronic medical records.

### Data analysis

Data were analyzed using IBM SPSS ver. 28.0 (IBM Corp., Armonk, NY, USA) at a statistical significance level of *p*<.05 for the following:

• General characteristics, disease-related characteristics, peripheral neuropathy symptoms, self-care ability, disturbances to daily life, and the QoL of the participants were analyzed based on real numbers, percentages, means, and standard deviations.

• Differences in the QoL of the participants according to general characteristics and disease-related characteristics were analyzed using the independent t-test and one-way analysis of variance, and post-hoc was conducted using the Scheffé test.

• The correlations between peripheral neuropathy symptoms, self-care ability, and disturbances to daily life were analyzed using Pearson correlation coefficients.

• The factors that affected QoL were identified by conducting multilinear regression analysis using the simultaneous input method.

• To identify the factors that impact the QoL of gynecological patients, multiple regression analysis was conducted by loading the three variables (CIPN symptoms, self-care ability, and disturbances to daily life) that showed significant differences in their correlations with QoL; also seven variables (age, level of education, monthly family income, regular exercise, experience with CIPN symptoms, the number of types of chemotherapy, and the number of treatments of chemotherapy) that showed statistically significant differences related to QoL, and multiple regression analysis was conducted using the simultaneous input method.

• The Durbin-Watson test value, which was calculated to test the assumption of the regression analysis, was 2.054, indicating no correlation between the independent variables. When multicollinearity was tested, the tolerance limit was .25–.83, which was larger than .1, and the variance inflation factor was 1.23–3.98, which did not exceed 10. These results indicated no problem related to multicollinearity.

## Results

### Differences in quality of life according to participants’ general characteristics and disease-related characteristics

The participants’ mean age was 56.38±8.59 years, and the highest number of participants was aged 50 to 59 years (n=59, 41.0%). A total of 123 participants (85.4%) were married, 81 (56.3%) were high school graduates, and 50 (34.7%) had a mean monthly income of 1 million Korean won (KRW; 1 million KRW is approximately 760 USD) to less than 3 million KRW. A total of 131 participants (91.0%) lived with their families, and 79 (54.9%) exercised regularly.

For disease-related characteristics, the highest percentage of participants had ovarian cancer at 70.1% (n=101), while 21 (14.6%) had endometrial cancer, 20 (13.9%) had cervical cancer, and 2 (1.4%) had another form of gynecological cancer (vaginal cancer or vulvar cancer). The highest proportion of participants answered that they first experienced CIPN after 1 to 3 chemotherapy treatments (n=99, 68.8%). A total of 57 participants (39.6%) had fewer than 6 months of experience with CIPN, while 40 (27.8%) had over 2 years of experience with CIPN. The mean number of different types of chemotherapy administered was 1.97±1.31; most of the participants were administered 1 type of chemotherapy (n=73, 50.7%). The mean number of chemotherapy treatments was 13.81±11.91, and the highest number of participants received treatment 6 to 10 times at 42 (29.2%). The mean cumulative amount of paclitaxel administered was 1,734.35±1,184.03 mg/m2, and 44 participants had a cumulative capacity of 1,000 to 1,499 mg/m2, making up the largest proportion of participants at 30.6%.

QoL showed significant differences according to age (F=12.64, *p*<.001), education level (F=6.15, *p*=.003), monthly family income (F=6.60, *p*=.002), regular exercise (t=3.99, *p*<.001), duration of CIPN experience (F=6.65, *p*=.002), the number of types of chemotherapy (F=6.10, *p*=.003), and the cumulative number of chemotherapy treatments (F=5.07, *p*=.008). Post-verification using the Scheffé test showed that the QoL was lower for participants aged 60 years and above compared to those below 60 years of age among the participants with a monthly family income of less than 1 million KRW compared to those with a monthly income of 3 million KRW or more. QoL was lower for those with over 24 months of experience with CIPN, those who were administered 3 or more types of chemotherapy, and those who received 11 or more chemotherapy treatments (Table 1).

### Degrees of quality of life, peripheral neuropathy symptoms, self-care ability, and disturbances to daily life of the participants

The mean score for QoL was low at 60.83±19.89, with the functional state (14.82±5.98) showing the lowest mean score. The mean score for CIPN symptoms among the participants was 42.53±19.73, with the highest score for sensory symptoms (30.93±13.93), followed by motor symptoms (11.60±7.37). The mean score for self-care ability was 144.33±28.79, and physical skills showed a relatively lower score compared to the other items. The mean score for disturbances to daily life was 56.24±34.09, with the highest mean score recorded for general activities (44.58±25.27) followed by manual dexterity (11.66±10.50) (Table 2).

### Correlations among main variables

Although CIPN among the participants had a moderate positive correlation with disturbances to daily life (r=.64, *p*<.001), it had a mild negative correlation with QoL (r=–.41, *p*<.001). While self-care ability had a mild negative correlation with disturbances to daily life (r=–.41, *p*<.001), it had a moderate positive correlation with QoL (r=.62, *p*<.001). Disturbances to daily life had a moderate negative correlation with QoL (r=–.62, *p*<.001) (Table 3).

### Factors affecting quality of life among participants

The QoL regression model of gynecological cancer patients undergoing chemotherapy was statistically significant (F=15.38, *p*<.001), and the explanatory power related to QoL in this model was 56.7%.

Four factors (self-care ability, intensity of disturbances to daily life, duration of CIPN symptoms, and regular exercise) were found to influence QoL. The factor with the greatest influence was self-care ability (β=.39, *p*<.001), followed by the intensity of disturbances to daily life (β=–.38, *p*<.001), the duration of CIPN symptoms (β=.14, *p*=.034), and regular exercise (β=–.13, *p*=.036). In other words, a lower QoL corresponded to a higher intensity of disturbances to daily life, a duration of CIPN symptoms of less than 6 months compared to 6 to 24 months, a lack of regular exercise, and a low self-care ability (Table 4). The number of different types of chemotherapy, monthly family income, intensity of CIPN symptoms, number of chemotherapy treatments, education level, and age did not have a significant impact.

## Discussion

This study was conducted to analyze data from gynecological cancer patients undergoing chemotherapy to understand the relationship between peripheral neuropathy symptoms, self-care ability, and disturbances to daily life, and identify the factors that affect QoL.

The QoL of gynecological cancer patients in this study was lower than in prior international and domestic studies conducted using the same instrument, which included QoL scores of 64.8 points [11], 78.8 points [28], 67.69 points [29], and 69.32 points [30]. The low QoL in this study was likely due to the high proportion of ovarian cancer patients at 70%; over 60% to 70% of whom had stage 3 or 4 cancer when they were diagnosed, as over 70% may experience recurrence even after treatment [15]. Moreover, the QoL was low for participants aged 60 years or above, those with a low education level (elementary/middle school graduates), and those with a monthly family income of less than 1 million KRW. Therefore, these factors should be considered when assessing the QoL of gynecological cancer patients, and different intervention programs should be devised according to age, education level, and income that are implemented based on the needs of participants when developing and implementing programs to improve QoL.

In this study, QoL had a negative correlation with peripheral neuropathy symptoms and disturbances to daily life and a positive correlation with self-care ability. This is similar to the results of a study that found that peripheral neuropathy symptoms and disturbances to daily life worsened as the number of neurotoxic anticancer agents administered increased [31] and a study that found that peripheral neuropathy symptoms and QoL had a negative correlation with each other among gynecological patients [11]. Peripheral neuropathy symptoms cause various physical dysfunctions in cancer patients that interfere with their activities of daily living and further decrease their QoL as well as self-care ability [32]. Although patients often attempt pharmacological and nonpharmacological interventions to alleviate their symptoms, no intervention methods or effective therapies to prevent peripheral neuropathy symptoms in cancer patients have been reported [33]. In accordance with studies that reported that exercise programs [34] and footbaths [35] improved blood circulation, this may be effective for mitigating peripheral neuropathy symptoms. More support is needed to develop effective interventions, and follow-up studies are needed on their effectiveness.

Self-care ability had the largest impact on QoL, followed by disturbances to daily life, the duration of CIPN symptoms, and regular exercise. In a prior study of general cancer patients, higher self-care ability corresponded to higher QoL [31], which is similar to the finding of another study that a higher level of self-care among colon cancer patients lead to proper symptoms management and improved QoL [19,21]. Self-care ability is a factor required of cancer patients to manage complications and return to normal life after treatment [32]. Thus, patients who have experienced peripheral neuropathy symptoms for a long time should periodically assess their ability to practice self-care when they go to the hospital. They should also be given sufficient information to improve their self-care ability, aas well as be offered programs that improve self-efficacy, form support groups, and take advantage of community resources [36].

The second factor that affected the QoL of the participants was the impact of disturbances to daily life. Patients who received high doses of paclitaxel, a neurotoxic anticancer agent, constituted a high-risk group for peripheral neuropathy symptoms and faced many restrictions to their activities of daily living since their symptoms lasted for longer periods. In addition, cancer patients experience physical symptoms caused by the treatment process, including pain, fatigue, and a lack of concentration, even after treatment ends [37], which has a negative influence on the QoL of gynecological cancer patients [5]. Therefore, to improve the QoL of the participants, it is also important to routinely measure the strength of disturbances to daily life during chemotherapy and prevent the exacerbation of the symptoms by offering patients rehabilitation therapy during treatment if necessary.

The duration of peripheral neuropathy symptoms was the third factor that affected QoL in this study. Peripheral neuropathy symptoms restrict motor and sensory functions and worsen the strength of disturbances to daily life, and symptoms worsen as the total amount of anticancer agents a patient is administered increases [31]. When peripheral neuropathy symptoms persist, patients experience physical dysfunction, disturbances to daily life, and deteriorated QoL [21,38]. Moreover, patients experience restrictions in their daily lives due to psychological problems such as anxiety and fear even after the completion of cancer treatment [6]. Thus, patients must have access to appropriate interventions from the beginning of chemotherapy to when they first notice peripheral neuropathy symptoms.

Regular exercise was the fourth factor that influenced the QoL of the participants. In a previous study of cancer patients [39], the QoL score was highest among those in the group with a high rate of physical activity, and the frequency at which patients participated in muscle exercises had a significant correlation with QoL [40]. Since the physical and psychological functions of cancer patients deteriorate over time, the implementation of a regular exercise program for patients to improve their physical function will likely improve their QoL. An exercise program should be devised for gynecological patients who have been hospitalized multiple times, and the importance of physical function should be reiterated routinely based on assessments of their muscle strength.

Based on this study, to improve the QoL of gynecological patients undergoing chemotherapy, an exercise-based intervention program should be developed to alleviate patients’ peripheral neuropathy symptoms and reduce the impact of disturbances to their daily lives. Moreover, correct information about the disease should be provided, self-efficacy improvement programs should be adopted, community support systems should be established, and accessibility to available resources should be increased to improve patients’ QoL. The QoL of gynecological cancer patients will likely improve through these methods, and follow-up studies should be conducted that consider when patients’ symptoms change by analyzing changes in peripheral neuropathy symptoms according to the cumulative amount of anticancer agents administered in the chemotherapy process that also specifies the time at which nursing interventions were conducted.

This study has limitations since the impacts of surgery, postoperative side effects, and recurrence on patients' QoL were not examined. Additionally, the results should be interpreted with caution since the data were from patients at a single hospital. Moreover, a total of 10 variables were analyzed using regression analysis in this study. When the sample size was calculated using G*Power 3.1.9 program with a significance level of .05, power of .85, a median effect size of .15, and 10 independent variables, the minimum sample size was 131 participants; however, when the power was set at .90, the minimum sample size was 147 participants. Thus, in future studies, the power should be increased by including more participants. This study makes meaningful contributions, however, as it showed that gynecological cancer patients who use neurotoxic anticancer agents have a high risk of peripheral neuropathy symptoms and identified the factors that influenced their QoL.

In conclusion, to improve the QoL of gynecological cancer patients, alleviation of peripheral neuropathy symptoms and promoting activities of daily living should be sought through the programs that improve physical function and the rehabilitation process. Furthermore, patients’ self-care ability should be enhanced through forming support groups and strenthening self-efficacy. In terms of follow-up studies, we recommend conducting longitudinal studies on the development of web-based programs to expand support systems and improve accessibility and developing programs to improve physical function.

## Figures and Tables

**Table 1.** Differences in quality of life according to participants’ characteristics (N=144)

Variable	Categories	Mean±SD or n (%)	Quality of life	
			Mean±SD	t or F (*p*)
*General characteristics*				
Age (year)	Range, 32–76	56.38±8.59		
	<50^a^	30 (20.8)	68.57±16.52	12.64 (<.001)
	50–59^b^	59 (41.0)	65.98±19.27	a, b>c^†^
	≥60^c^	55 (38.2)	51.07±18.52	
Marital status	Single	13 (9.0)	63.38±18.85	0.19 (.829)
	Married	123 (85.4)	60.74±20.04	
	Others	8 (5.6)	58.00±21.26	
Education level	≤Middle school^a^	16 (11.1)	50.31±20.71	6.15 (.003)
	High school^b^	81 (56.3)	58.74±19.46	a<c^†^
	≥College^c^	47 (32.6)	68.00±18.24	
Monthly family income (KRW)	<1 milion^a^	49 (34.0)	53.92±19.77	6.60 (.002)
	1–3 milion^b^	50 (34.7)	60.88±20.85	a<c^†^
	≥3 milion^c^	45 (31.3)	68.29±16.28	
Living with family	Yes	131 (91.0)	60.85±19.75	–.040 (.968)
	No	13 (9.0)	60.62±22.10	
Regular exercise	Yes	79 (54.9)	66.53±18.41	3.99 (<.001)
	No	65 (45.1)	53.89±19.55	
*Disease-related characteristics*				
Type of cancer	Ovarian	101 (70.1)	60.06±18.53	0.85 (.469)
	Endometrial	21 (14.6)	60.90±24.02	
	Cervical	20 (13.9)	64.05±22.77	
	Others (vaginal/vulvar)	2 (1.4)	82.00±18.39	
First incidence of CIPN	1–3	99 (68.8)	60.64±18.94	0.30 (.744)
	4–6	28 (19.4)	59.50±21.11	
	≥7	17 (11.8)	64.12±23.90	
Duration of CIPN (month)	<6 ^a^	57 (39.6)	62.23±20.90	6.65(.002)
	6–23^b^	47 (32.6)	66.68±18.80	a, b>c^†^
	≥24^c^	40 (27.8)	51.95±16.77	
Types of chemotherapy drugs	Range, 1–7	1.97±1.31		
	1^a^	73 (50.7)	66.10±19.41	6.10 (.003)
	2^b^	32 (22.2)	58.00±21.36	a, b>c^†^
	≥3^c^	39 (27.1)	53.28±16.87	
Number of chemotherapy treatments	Range, 4–60	13.81±11.91		
	1–5^a^	37 (25.7)	61.32±20.60	5.07 (.008)
	6–10^b^	42 (29.2)	68.05±17.49	a, b>c^†^
	≥11^c^	65 (45.1)	55.88±19.75	
Cumulative amount of paclitaxel (mg/m^2^)	Range, 700–7,715	1,734.35±1,184.03		
	700–999	39 (27.1)	61.26±21.20	0.99 (.374)
	1,000–1,499	44 (30.6)	63.86±19.15	
	≥1,500	61 (42.3)	58.36±19.56	

CIPN: Chemotherapy-induced peripheral neuropathy; KRW: Korean won (1 million KRW is approximately 760 USD).

† Scheffé test.

**Table 2.** Levels of quality of life, CIPN symptoms, self-care ability, and disturbances to daily life (N=144)

Variable	Categories	Mean±SD	Possible range	Data range
Quality of life		60.83±19.89	0–108	5–106
	Psychological state	14.89±4.82	0–24	0–24
	Physical state	15.91±7.34	0–28	0–28
	Social state	15.21±6.42	0–28	0–28
	Functional state	14.82±5.98	0–28	0–28
CIPN symptoms		42.53±19.73	0–90	2–90
	Sensory symptoms	30.93±13.93	0–60	2–58
	Motor symptoms	11.60±7.37	0–30	0–30
Self-care ability		144.33±28.79	32–192	46–190
	Perception of self-monitoring	5.20±0.96	1–6	2–6
	Attention to self-management	14.83±2.43	3–18	6–18
	Cognitive aspects of self-cares	50.48±10.27	6–66	11–66
	Judgment and decision-making process	18.33±4.07	4–24	4–24
	Information-seeking behaviors	17.10±3.50	4–24	5–24
	Physical skills		9–54	9–54
Disturbances to daily life		56.24±34.09	0–140	0–140
	General activities	44.58±25.27	0–100	0–100
	Manual dexterity	11.66±10.50	0–40	0–40

CIPN: Chemotherapy-induced peripheral neuropathy.

**Table 3.** Relationships among study variables (N=144)

Variable	r (*p*)		
	Quality of life	CIPN symptoms	Self-care ability
Quality of life	1		
CIPN symptoms	–.41 (<.001)	1	
Self-care ability	.62 (<.001)	–.16 (.055)	1
Disturbance of daily life	–.62 (<.001)	.64 (<.001)	–.41 (<.001)

CIPN: Chemotherapy-induced peripheral neuropathy.

**Table 4.** Factors influencing quality of life (N=144)

Variable	B	SE	β	t (*p*)
(Constant)	35.22	12.91		3.37 (.001)
Regular exercise^†^	–5.00	2.36	–.13	–2.12 (.036)
Number of types of chemotherapy	–2.67	1.67	–.18	–1.60 (.113)
Monthly family income (KRW)^†^				
1–3 million	2.87	2.98	.07	0.97 (.336)
>3 million	1.08	3.13	.04	0.58 (.556)
Number of chemotherapy treatments	0.08	0.18	.05	0.45 (.655)
Education level^†^				
≤Middle school	0.64	3.86	.01	0.17 (.868)
≥College	1.28	2.69	.03	0.48 (.635)
Age (year)	0.07	0.16	.03	0.44 (.662)
Self-care ability	0.27	0.05	.39	5.65 (<.001)
Disturbances to daily life	–0.22	0.05	–.38	–4.51 (<.001)
Duration of peripheral neuropathy symptoms^†^ (month)				
6–23	6.04	2.82	.14	2.14 (.034)
≥24	2.14	4.2	.05	0.51 (.611)
CIPN symptoms	–0.06	0.08	–.06	–0.85 (.400)
R²=60.6, adjusted R²=56.7, F(*p* )=15.38(<.001)				

CIPN: Chemotherapy-induced peripheral neuropathy; KRW: Korean won (1 million KRW is approximately 760 USD).

† References were duration of peripheral neuropathy symptoms (<6 months), regular exercise (yes), monthly family income (<1 million KRW), and education level (high school).

## References

[1] Sung H, Ferlay J, Siegel RL, Laversanne M, Soerjomataram I, Jemal A (2021). Global Cancer Statistics 2020: GLOBOCAN estimates of incidence and mortality worldwide for 36 cancers in 185 countries. CA Cancer J Clin.

[2] http://kosis.kr/statHtml/statHtml.do?orgId=117&tblId=DT_117N_A00021&conn_path=I2.

[3] Oh PJ, Kim YL (2018). Effectiveness of non-pharmacologic interventions in chemotherapy induced peripheral neuropathy: a systematic review and meta-analysis. J Korean Acad Nurs.

[4] Jung JY, Lee JM, Kim MS, Shim YM, Zo JI, Yun YH (2018). Comparison of fatigue, depression, and anxiety as factors affecting posttreatment health-related quality of life in lung cancer survivors. Psychooncology.

[5] Shin N, Kim J (2017). Experience of chemotherapy in ovarian cancer patients. Asian Oncol Nurs.

[6] Park JA, Hong JY (2017). Factors influencing quality of life in adult cancer patients: the sixth Korea National Health and Nutrition Examination Survey (KNHANES VI-2), 2014. J Korea Acad-Industr Coop Soc.

[7] Kim HS, Yi M (2015). Factors influencing quality of life in multiple myeloma patients. Asian Oncol Nurs.

[8] Ryu YM, Yi M (2013). The factors influencing quality of life in women with breast cancer. Asian Oncol Nurs.

[9] Yu SY, Kim JH (2017). Lower limb lymphedema and quality of life in gynecologic cancer patients. Asian Oncol Nurs.

[10] Yu SY, Nho JH (2015). Influence of sleep disturbance and depression on quality of life in ovarian cancer patients during chemotherapy. Asian Oncol Nurs.

[11] Jeong JH, Nho JH, Kim GS, Lee YE, Yu SY, Lee HJ (2013). Characteristics and quality of life in gynecologic cancer patients with chemotherapy-induced peripheral neuropathy. Korean J Women Health Nurs.

[12] Park JS, Oh YJ (2012). Factors influencing on quality of life in gynecological cancer patients. Korean J Adult Nurs.

[13] Suh DH, Kim M, Kim K, Kim HJ, Lee KH, Kim JW (2017). Major clinical research advances in gynecologic cancer in 2016: 10-year special edition. J Gynecol Oncol.

[14] Staff NP, Grisold A, Grisold W, Windebank AJ (2017). Chemotherapy-induced peripheral neuropathy: a current review. Ann Neurol.

[15] Berek JS, Renz M, Kehoe S, Kumar L, Friedlander M (2021). Cancer of the ovary, fallopian tube, and peritoneum: 2021 update. Int J Gynaecol Obstet.

[16] Choe YH, Kim SH, Oh HS, Seo WS, Lee SH (2020). Chemotherapy-induced peripheral neuropathy in patients with breast cancer: Associated factors and impact on health-related quality of life. Asian Oncol Nurs.

[17] Kim KY, Lee SH, Kim JH, Oh PJ (2015). Disturbance in ADL from chemotherapy-induced peripheral neuropathy and quality of life in cancer patients: the mediating effect of psychological distress. J Korean Acad Nurs.

[18] Oh PJ, Choi ES, Lee J (2019). The experience of chemotherapy-induced peripheral neuropathy in people with cancer. Asian Oncol Nurs.

[19] Yin L, Fan L, Tan R, Yang G, Jiang F, Zhang C (2018). Bowel symptoms and self-care strategies of survivors in the process of restoration after low anterior resection of rectal cancer. BMC Surg.

[20] Bae KR, Im YS, Noh GO, Son Y, Seo HG (2017). Relationships among hope, self-care agency and quality of life of female oncology patients with lymphedema. Asian Oncol Nurs.

[21] Ose D, Winkler EC, Berger S, Baudendistel I, Kamradt M, Eckrich F (2017). Complexity of care and strategies of self-management in patients with colorectal cancer. Patient Prefer Adherence.

[22] Cella DF, Tulsky DS, Gray G, Sarafian B, Linn E, Bonomi A (1993). The functional assessment of cancer therapy scale: development and validation of the general measure. J Clin Oncol.

[23] Kim H, Yoo HJ, Kim YJ, Han OS, Lee KH, Lee JH (2003). Development and validation of Korean functional assessment cancer therapy-general (FACT-G). Korean J Clin Psychol.

[24] Tofthagen CS, McMillan SC, Kip KE (2011). Development and psychometric evaluation of the chemotherapy-induced peripheral neuropathy assessment tool. Cancer Nurs.

[25] Hwang WH, Park GJ (2014). Peripheral neuropathy in cancer patients undergoing chemotherapy. J Wholistic Nurs Sci.

[26] Geden E, Taylor S (1991). Construct and empirical validity of the Self-As-Carer Inventory. Nurs Res.

[27] Jung Y (1993). The relationship between self-care agency and quality of life of cancer patients. J Korean Acad Adult Nurs.

[28] Hung HW, Liu CY, Chen HF, Chang CC, Chen SC (2021). Impact of chemotherapy-induced peripheral neuropathy on quality of life in patients with advanced lung cancer receiving platinum-based chemotherapy. Int J Environ Res Public Health.

[29] Kim H, Park H (2018). Chemotherapy induced peripheral neuropathy, sleep and quality of life among patients with gastric cancer receiving chemotherapy. J Korean Acad Fundam Nurs.

[30] Kim GE, Song JE, You MA, Park JH (2022). Symptom experience, social support, and quality of life in patients with hematologic malignancies undergoing chemotherapy. Asian Oncol Nurs.

[31] Kim JH, Lee KM, Jeon MJ, Seol ME, Lee SH, Park J (2013). Symptom and interference of activities of daily living of chemotherapy-induced peripheral neuropathy in patients receiving taxanes and platinums. Asian Oncol Nurs.

[32] Kim MJ, Shin YS (2021). Relationship between health literacy and self-care behavior in patients with stomach cancer after gastrectomy: Mediating effects of subjective health status and specific self-efficacy. Korean J Adult Nurs.

[33] Woo IS (2015). Recent updates on chemotherapy-induced peripheral neuropathy. Korean J Intern Med.

[34] Choe YH, Kim DH (2021). Effects of exercise on chemotherapy-induced peripheral neuropathy: a systematic review and meta-analysis. Korean J Adult Nurs.

[35] Kim HA, Lim KH (2021). Effects of foot bath therapy on peripheral neuropathy, sleep disorder, and fatigue in gynecologic patients with cancer undergoing chemotherapy. Korean J Adult Nurs.

[36] Lim JW, Sohn J, Back J (2020). A study on developing a self-efficacy enhancement program and evaluating its effects for breast cancer survivors. Korean J Soc Welf.

[37] Kim SY (2020). The experience of cancer survivor's return to everyday life. J Korea Acad-Industr Coop Soc.

[38] Bao T, Basal C, Seluzicki C, Li SQ, Seidman AD, Mao JJ (2016). Long-term chemotherapy-induced peripheral neuropathy among breast cancer survivors: prevalence, risk factors, and fall risk. Breast Cancer Res Treat.

[39] Min JH, Kim JY, Lee J, Jeon YJ (2020). The association between physical activity domain and quality of life among Korean cancer patients and survivors: The Korean National Health and Nutrition Examination Survey (KNHNES) 2014-2017. Korean J Phys Educ.

[40] An KY, Kang DW (2018). The association between resistance exercise frequency, muscular strength, and health-related quality of life in Korean CANCER PATIENTS: The Korea National Health and Nutrition Examination Survey (KNHANES) 2014-2016. Korean J Phys Educ.

